# Measuring concentration and diffusivity within biomolecular condensates using calibration-free scanning fluorescence correlation spectroscopy

**DOI:** 10.1039/d5sc05592j

**Published:** 2025-11-19

**Authors:** Prerit Mathur, Marcell Papp, Katarzyna Makasewicz, Paolo Arosio, Andrew J. deMello, Stavros Stavrakis

**Affiliations:** a Institute for Chemical and Bioengineering, Department of Chemistry & Applied Biosciences, ETH Zürich 8093 Zürich Switzerland andrew.demello@chem.ethz.ch stavros.stavrakis@chem.ethz.ch

## Abstract

There is a growing consensus that cells can regulate biochemical activity through membrane-less organelles, also known as biomolecular condensates. Unfortunately, the mechanisms underlying the interplay between phase separation and biochemical reactions are still unclear. Since biochemical reactions depend strongly on the local concentrations and diffusivities of molecules in the dense phase, accurately characterizing these parameters is essential for understanding biochemical regulation within phase-separated condensates. Fluorescence correlation spectroscopies can measure these properties but are limited by their need for calibration standards. Here, we present a calibration-free method based on temporal line scan fluorescence correlation spectroscopy and sinusoidal scan fluorescence correlation spectroscopy to quantify concentrations and diffusivities of molecules in the dilute and dense phases. We showcase the potential of the approach by measuring the full phase diagram of the intrinsically disordered region of the DEAD-box protein Ddx4, as well as the diffusivities of recruited client molecules in the dense phase. We show that the diffusivity of different client molecules decreases as their concentration in the dense phase increases. Such a drastic decrease in diffusivities may explain the stability of certain aggregation-prone proteins in the dense phase despite their high local concentrations.

## Abstract 2

Line scanning fluorescence correlation spectroscopy is a calibration-free measurement technique that enables the mapping of phase diagrams of protein condensates and the quantification of peptide recruitment within condensates.

## Graphical abstract

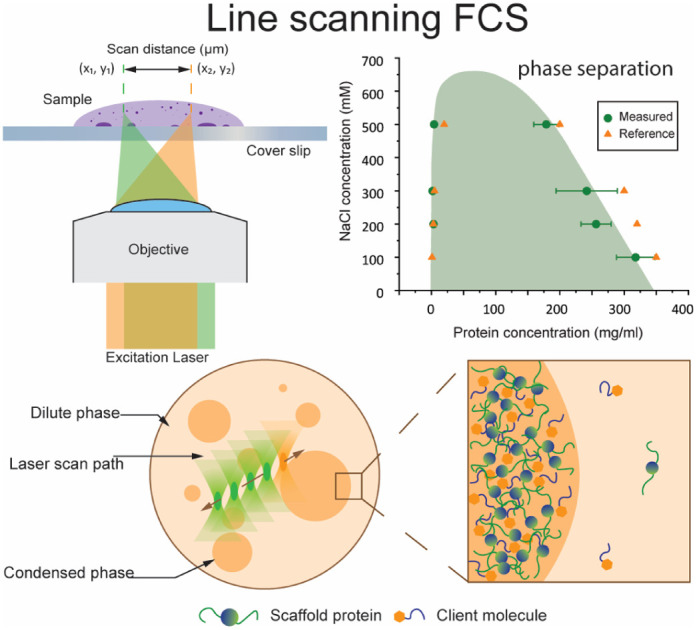

## Introduction

**Fig. 1:**
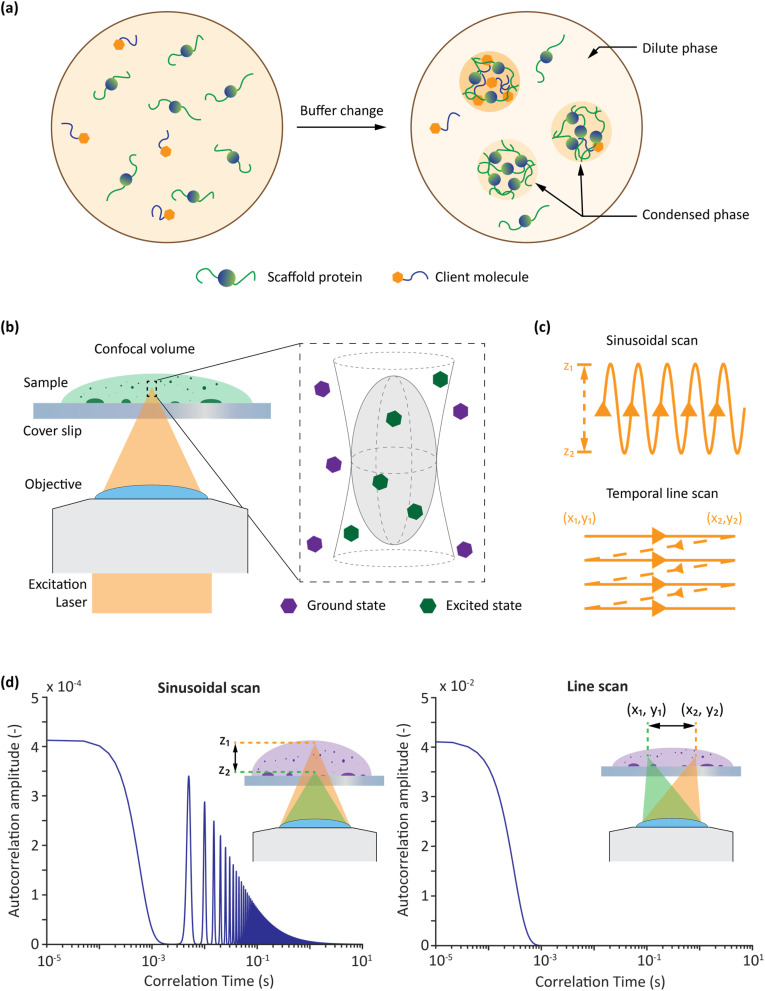
Overview of phase separation, FCS and scanning FCS. (a) Phase separation is induced in protein samples by changing buffer conditions and forming distinct dense and dilute phases. (b) FCS is used to probe a defined volume in both the dilute and dense phases. (c) A laser is scanned across the sample in bespoke sinusoidal and linear patterns to perform calibration-free FCS. (d) Representative autocorrelation curves associated with sinusoidal and line scanning FCS, with the motion of the laser path shown in the insets.

In addition to membrane-bound compartments, it is now recognized that cells can coordinate biochemical activity *via* membrane-less organelles, also known as biomolecular condensates. Biomolecular condensates are complex viscoelastic entities formed *via* the phase separation of proteins and nucleic acids and comprising a dense phase surrounded by a dilute phase.^1–3^ The molecules that drive phase separation, commonly termed “scaffolds”, form condensates that can recruit “client” molecules into their interior.^4,5^ By regulating the local concentration of clients in both space and time, such condensates can modulate enzymatic reactions and aggregation events.^6–11^ A germane example, in this regard, is the ability of certain heterotypic scaffold condensates to suppress aggregation of aggregation-prone proteins recruited in their interior, despite their high local concentrations (Fig. 1a). This effect, known as heterotypic buffering, involves a competition between scaffold–client interactions that drive recruitment, and client–client interactions that lead to aggregate formation.^12,13^ The mechanism is highly relevant, since it may control the ability of condensates to suppress aberrant aggregation of misfolded proteins recruited into stress granules in response to stresses such as heat shock or starvation.^13,14^ Indeed, it has recently been shown that *in vitro* condensates can suppress fibril formation of the Abeta42 (Aβ42) peptide, despite its high aggregation propensity and the millimolar concentrations observed in condensate interiors.^9–11^ Whilst the solution phase peptide forms amyloids within hours at nanomolar concentrations, upon recruitment into condensates the peptide remains in the monomeric form for several hours.^15^ Another relevant example is fibril formation of the nuclear protein TDP-43 triggered by de-mixing within stress granules. Under non-oxidative stress, mislocalized TDP-43 is homogeneously dispersed within stress granules. Heterotypic interactions with the scaffold prevent the highly concentrated TDP-43 from condensing and aggregating.^16^

To understand how protein condensation can regulate biochemical activities, including protein aggregation, it is essential to characterize the emergent properties of biomolecular condensates. Such properties include the concentration of molecules in the dense and dilute phases, as well as the diffusivity of scaffold and client molecules within the dense phase. Changes in diffusivity can potentially lead to mass transfer limitations, which inhibit aggregation processes and reaction rates. Indeed, even for reactions involving small molecules, diffusion limitations can be present, since enzymatic reactions often occur on time-scales similar to diffusion events.^9^

Over the past decade, a variety of computational, optical and microfluidic methods have been used to characterise the emergent properties of condensates and determine phase diagrams upon variation of salt, temperature and pH.^17,18^ Among these, fluorescence correlation spectroscopy (FCS) has often been used to measure the concentrations of molecules in both dilute and dense phases.^19–21^ FCS analyses fluctuations in fluorescence intensity within a defined volume to report the dynamic motion of a molecule and infer properties such as diffusivity and concentration (Fig. 1b).^22^ For example, FCS has been used to study binding interactions,^23^ protein adsorption,^24^ enzyme diffusion^25^ and drug nanocarriers.^26^ Despite its power, traditional point-based FCS has a well-known limitation, namely the need to calibrate the confocal volume using a solution that is optically similar to the (unknown) sample to be measured. This requirement does not pose significant problems when probing dilute aqueous solutions, as aqueous calibration standards (with well-defined diffusivities) have been extensively characterised in the literature.^27^ However, the need for calibration is far more problematic when the standards used for calibration have different optical properties (with respect to refractive index and optical saturation of the fluorophore moiety^28,29^) than the sample of interest. A change in refractive index can change both the apparent concentration and diffusivity of the sample being measured.^30,31^ Changes in refractive index between the immersion medium and the sample cause optical aberrations that distort the confocal detection volume in point FCS measurements. These aberrations primarily affect the axial dimension of the detection volume, causing its elongation and deformation, which directly impacts the volume calibration and leads to erroneous estimates of the molecular concentration.^30^ At the same time, lateral distortions of the detection volumes can also occur, influencing the effective lateral beam waist and thus altering the measured diffusion times used to calculate diffusion constants.^31^ This dual effect compromises both concentration and diffusion measurements, making point FCS unreliable under high refractive index mismatch conditions. This is particularly relevant when considering biomolecular condensates, since the dense phase will normally have a very different refractive index when compared to the calibration sample.^32^ Moreover, the curvature of condensates can also distort the confocal volume, as a curved surface can act as an additional lens introducing unexpected aberrations. The combined effects of both issues lead to inaccuracies in the estimation of the confocal volume by traditional FCS, leading to significant inaccuracies when measuring the concentrations of unknown samples.^28,33^ To overcome these problems, different calibration-free scanning FCS (sFCS) methods have been proposed.^27,34–36^ Each of these methods involves optical scanning over a characteristic dimension (length or radius) at a characteristic speed (or frequency). Using known scan parameters, the confocal volume and therefore the unknown concentration can be calculated directly from autocorrelation functions (ACFs) recorded in both space and time, without the need for calibration.^34^ Furthermore, by sampling a region rather than a single point, sFCS also reduces artifacts resulting from bleaching or phototoxicity.^37^

Here we use a combination of sinusoidal scanning FCS (sineFCS),^19^ and our newly developed temporal line scanning FCS (tl-FCS) to measure the concentrations of both slowly and rapidly diffusing species. This allows the analysis of both dense and dilute phases in a phase separated sample (Fig. 1c and d). Previous studies have suggested that a significant increase in laser scanning speed would be required for any line scanning FCS method to effectively capture fast diffusion dynamics.^38^ This increase in speed is considered necessary because a line scan is most commonly performed by analysing a series of independent lines, which correspond to different time points. This approach, which involves treating a line as being inherently discontinuous (with defined start and end points), limits time resolution. In tl-FCS, we treat the line scan as a piecewise continuous function, enabling the effective measurement of fast processes (down to 100 ns). Significantly, tl-FCS requires no additional hardware and can be implemented on any standard confocal laser scanning microscope by simply introducing an additional data analysis pipeline. By combining both scanning FCS techniques, our platform can analyse diffusion timescales between 100 ns and 1 s, without the need for calibration, enabling the measurement of concentrations and diffusivities in both dilute and dense phases of phase separated protein solutions.

We validate our two scanning FCS methods by measuring the phase diagram of the intrinsically disordered region of the DEAD-box protein Ddx4(1-236). We analyse individual condensates and uncover a broad distribution of properties within the same sample. Ddx4(1-236) was selected as a model protein due to its well-established role as a core component of germ granules in germline cells. These membraneless organelles are critical for RNA regulation and transposon silencing during germ cell development.^39^ The intrinsically disordered N-terminal region of Ddx4, which encompasses the first 236 amino acids, drives phase separation and formation of liquid-like germ granules through multivalent weak interactions, primarily electrostatic in nature. This property makes it an ideal candidate for studying the biophysical and biochemical mechanisms of condensate formation, RNA organization, and its regulation within germ cells.^40^

We further use our method to measure the concentration and diffusivity of client molecules recruited within condensates. Specifically, we focus on the Aβ42 peptide and Atto565 dye as clients recruited into condensates formed by Ddx4(1-236) and a chimeric protein based on the arginine–glycine rich (RGG) region of Laf1.^41^ The RGG construct was chosen because of its widespread use as a model system to investigate phase-separated condensates.^42^

Importantly, data indicate a decrease in the diffusivity of the client molecule with increasing concentration of the client within the dense phase. These results are important for understanding how condensates can suppress the oligomerization and aggregation of aggregation-prone peptides in their interior, despite their high local concentration.

## Results and discussion

### Temporal line scanning FCS

tl-FCS measurements were performed on a Nikon C2 confocal scanner, while sineFCS was performed by introducing an additional sinusoidal scanning lens in the optical path. The requirements for scanning FCS are different depending on the scanning modality chosen. In general, all FCS data analysis followed a two-step process. First, the photon signal was corrected for photobleaching effects, and the dead time of the sensor and the ACF were then calculated from the corrected signal (SI Text 1). Second, the ACF was fitted to the appropriate theoretical model (SI Text 2). Signal correction and ACF calculation steps were common to all FCS data collected, with the final data fitting depending on which scanning modality was used. SI Text 2 fully describes the theory and, where required, the optical path changes implemented for point FCS, sineFCS and tl-FCS.

**Fig. 2:**
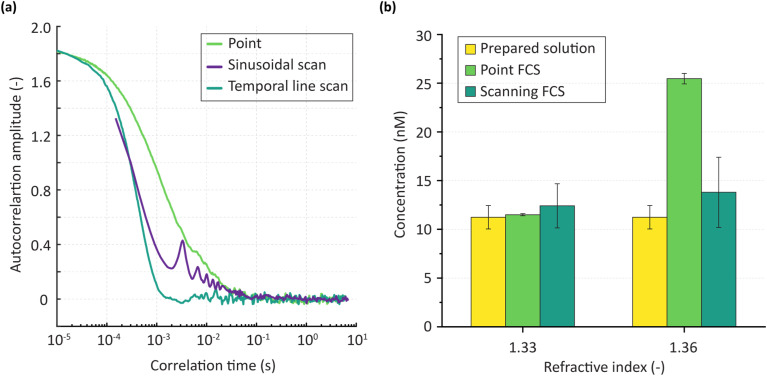
Comparison between scanning FCS methods and standard point FCS. (a) ACF of scanning FCS methods in agreement with point FCS for a dilute aqueous solution of 20 nm fluorescent beads. The point ACF envelops both scanning FCS as is expected theoretically. (b) Changing refractive index of a solution shows measurement errors in point FCS. Aqueous solution shows agreement in concentration measurements while sucrose solution with a higher refractive index shows large errors in point measurement. Error bars indicate standard deviation of multiple (*n* = 3) measurements.

We first validated the performance of our scanning FCS methods (compared to traditional point FCS) by measuring a low concentration aqueous colloidal dispersion. Specifically, we compared the ACFs extracted using point FCS, sineFCS and tl-FCS from a solution of 20 nm diameter fluorescent polystyrene beads in PBS buffer (Fig. 2a). As the bead solution mimics a dilute aqueous sample, we used a 5.6 nM Atto565 solution as the calibration standard for point FCS. The raw correlation curves extracted using each method have the same *y*-intercept, confirming that the same number of molecules are present within the confocal volume and that concentrations are identical (Fig. 2a). Significantly, the three methods yield consistent correlation times as shown in Fig. 2a. We note that, as expected, the point scan ACF envelops both the peaks generated by the sinusoidal scan ACF and the ACF generated by tl-FCS (eqn (6), (7) and (9) in SI). Additionally, the correlation time of tl-FCS is dependent on the laser scanning speed (Fig. S1). Overall, the concentration and diffusivity values calculated by all three methods provided consistent results (1.13 ± 0.11 nM, 1.14 ± 0.28 nM and 1.38 ± 0.36 nM concentration and 19.18 ± 0.73 µm^2^ s^−1^, 16.94 ± 2.81 µm^2^ s^−1^ and 16.52 ± 2.34 µm^2^ s^−1^ diffusivity for point, sinusoidal and line measurements respectively), therefore validating the use of our scanning FCS method in dilute aqueous samples.

**Fig. 3:**
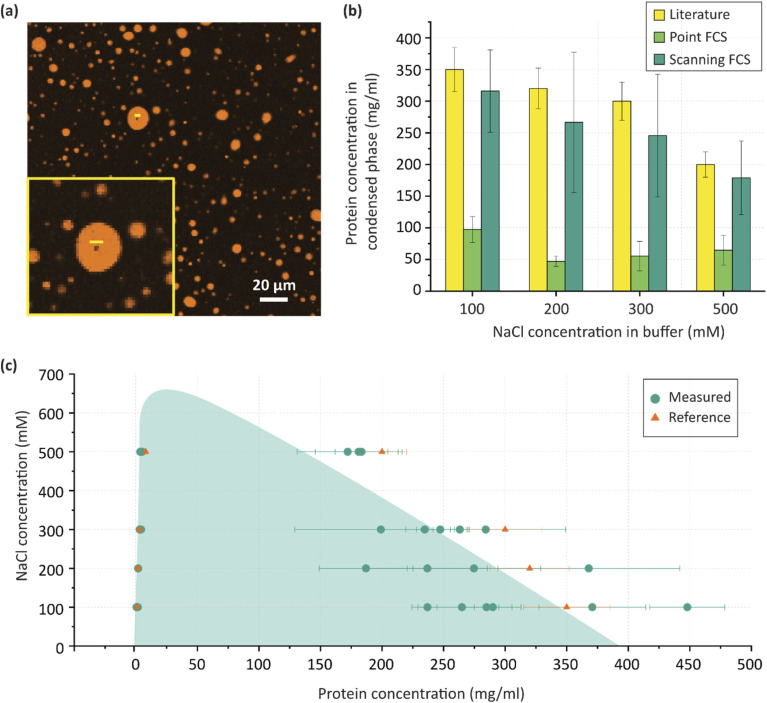
Phase diagram of Ddx4-Atto565 measured with FCS. (a) Fluorescence confocal microscopy image of a phase separated sample at 200 µM Ddx4 in a sodium phosphate buffer. A yellow line overlay on a droplet shows a typical tl-FCS line scan path. Inset shows the scan line in detail. (b) Comparison of protein concentration in the dense phase measured by point FCS, scanning FCS and reference values reported in literature. Error bars represent the standard deviation of multiple (*n* = 3 to 6) measurements. (c) Phase diagram of Ddx4 as a function of salt concentration. Multiple points represent measurement of individual condensates. The shaded region is presented as a visual aid. Error bars represent standard error in each measurement.

Next, we aimed to assess the accuracy of scanning FCS methods for samples having a higher refractive index of 1.36. A refractive index of 1.36 was chosen, as this value falls within the range reported for concentrated protein solutions.^32^ This was achieved using two 11.2 nM Atto565 solutions, one aqueous with a refractive index of 1.33 and the other containing 20% by weight of sucrose, having a refractive index of 1.36.^43^ For point FCS measurements, a calibration solution of 5.6 nM Atto565 was again used. While both point FCS and scanning FCS accurately measured the expected concentration (11.4 ± 0.25 nM and 12.4 ± 2.10 nM, respectively) in the aqueous solution, point FCS overestimated concentration by a factor of 2 (24.1 ± 0.40 nM) when the refractive index was increased to 1.36. In contrast, scanning FCS maintained accuracy, reporting a concentration of 13.8 ± 3.60 nM (Fig. 2b). This observation highlights the need to carefully choose the calibration sample for point FCS, which is especially challenging when analysing samples with unknown characteristics.^20^ To emphasise the importance of this issue, we next measured a highly concentrated protein sample by inducing phase separation of 200 µM Ddx4(1-236) in sodium phosphate buffer (Fig. 3a) and comparing results with previous values reported in the literature.^44^ Concentration measurements in the dense phase were performed using both point FCS (using a 5.6 nM aqueous Atto565 calibration solution) and sineFCS. Comparison of the measured concentrations with literature values (Fig. 3b) reveals a notable discrepancy between point FCS-derived and reported concentrations, whereas values obtained using sineFCS show excellent agreement with the literature. It should be noted that error bars associated with sineFCS measurements are relatively large as they take account of intrinsic protein heterogeneity and the variability of concentration among different condensates. These findings again underscore the limitations of point FCS, which, despite its utility when probing dilute solutions, fails to provide reliable measurements in the high refractive index environments that are typical of dense protein phases. Conversely, scanning FCS provides reliable measurements in both scenarios, without the need for calibration.

### Phase separation of scaffold molecules

We then applied a combination of tl-FCS and sineFCS to generate the phase diagram of Ddx4(1-236) (Fig. 3c), for fast- and slow-diffusing species. We induced phase separation of 200 µM Ddx4(1-236) in a sodium phosphate buffer at room temperature (20 °C) using different NaCl concentrations and measured the protein concentration in both the dilute phase (*c*_s_) and the dense phase (*c*_d_). As shown in Fig. 3c, our measurements were in good agreement with previously published values.^44^ Specifically, for a NaCl concentration of 100 mM, we measured *c*_d_ to be 315.9 ± 65.0 mg ml^−1^ (12.20 ± 2.50 mM), whilst *c*_s_ was measured to be 0.90 ± 0.12 mg ml^−1^ (34.70 ± 4.48 µM). These data indicate a more than two order-of-magnitude increase of protein concentration in the dense phase. Importantly, sineFCS further allows for individual droplet concentration measurements, revealing intra-droplet heterogeneity (Fig. 3c). We observed that the dense phase concentration can vary by as much as a factor of two depending on which condensates are measured. Even larger heterogeneities in phase separation have been observed in the literature. For instance Jawerth *et al.*^45^ described pronounced differences in condensate concentration emphasizing the dynamic and heterogeneous nature of phase-separated compartments. In addition to concentration, tl-FCS and sineFCS also allow independent measurement of diffusivity. We observed significant heterogeneity in the diffusivity among condensates within the same sample at each tested salt concentration (Fig. S2).

### Partitioning and diffusivity of client molecules in the dense phase

**Fig. 4:**
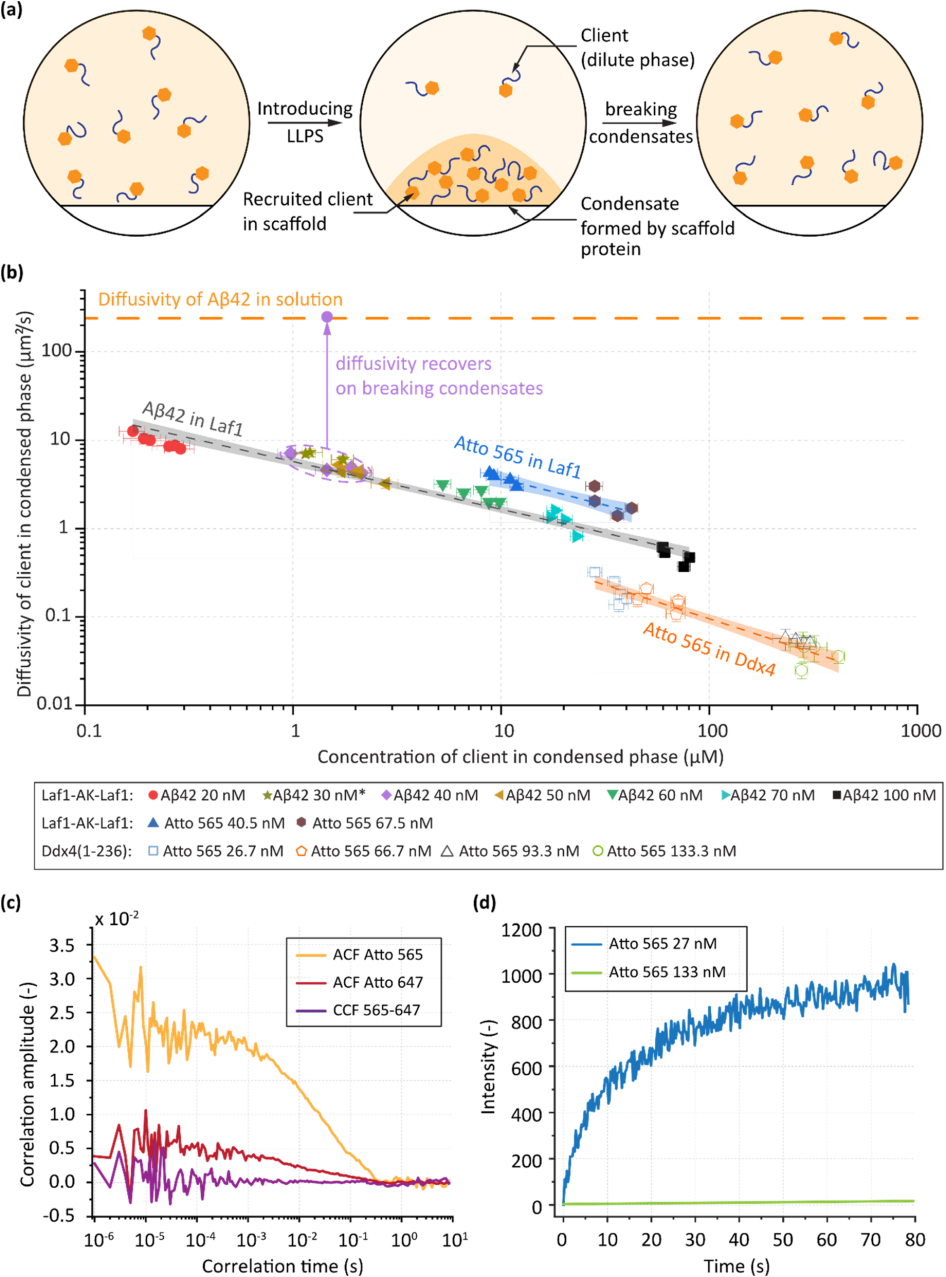
Variations of the properties of clients in a scaffold as a function of recruited concentration. (a) Schematic showing the recruitment of a client into the condensed phase formed by a scaffold. The client partitions preferentially inside the condensate. (b) Variation of the diffusivity of the client in the condensed phase as a function of client concentration for Aβ42-Atto565 and Atto565 being recruited into Laf1-AK-Laf1 or Ddx4 droplets. The straight lines represent a power law fit of diffusivity with concentration and the shaded regions represent the 95% confidence interval. On breaking condensates by increasing salt in the solution, the measured diffusivity of the recovered Aβ42-Atto565 is the same as Aβ42-Atto565 in dilute solution. Error bars represent standard error in each measurement. (c) FCCS of Aβ42-Atto488 and Aβ42-Atto647 recruited in Laf1-based condensates shows no cross correlation, indicating no oligomerisation. Both Aβ42-Atto488 and Aβ42-Atto647 were added to phase separated Laf1-based condensates simultaneously. (d) Representative FRAP curves of Atto565 recruited into Ddx4-based condensates shows large differences in diffusivity depending on the added total concentration of the dye.

After demonstrating the utility of our method in characterizing the concentration and diffusivity of scaffold proteins inside the dense phase, we next measured the properties of client molecules recruited into the dense phase (Fig. 4a). As clients, we chose Atto565 and Aβ42 labelled with Atto565 as mimics for a small molecule and a larger macromolecule, respectively. We measured their properties in condensates formed by either Ddx4(1-236) or Laf1-AK-Laf1, a chimeric protein.^15,41^ This protein contains intrinsically disordered domains of Laf1 linked to the enzyme adenylate kinase (AK) and is referred to as Laf1-AK-Laf1.

We first analysed Aβ42 labelled with Atto565 recruited into Laf1-AK-Laf1 condensates. Previous studies have shown that the recruitment of Aβ42 inside these condensates strongly inhibits its aggregation despite local increases in concentration of several orders of magnitude.^15^ We note that these experiments were performed using unlabelled peptide, demonstrating that the recruitment inside the condensates and the inhibition of aggregation are independent of fluorophore presence.^15^ Our FCS analysis confirmed previous results, indicating that essentially all Aβ42 is recruited inside the condensates, with the concentration in the dilute phase being below the detection limit. Introducing labelled Aβ42 in solution, at concentrations between 20 nM and 100 nM, we measured a client concentration in the dense phase ranging from 0.2 µM to 100 µM; a variation of three orders of magnitude with respect to the initial protein concentration.

Diffusivity measurements of Aβ42 at a total added concentration of 20 nM showed a decrease with respect to values measured in dilute solution of one order of magnitude (from 250 µm^2^ s^−1^ to 10 µm^2^ s^−1^) (Fig. 4b). Significantly, diffusivity decreased with increasing peptide concentration within the dense phase, reaching a value of as low as 0.5 µm^2^ s^−1^; three orders of magnitude lower than diffusivity values measured in dilute solution. Additionally, we observed considerable heterogeneity in both concentration and diffusivity between individual condensates under the same conditions (Fig. 4b). Interestingly, when plotting the normalised diffusivity within the condensed phase (relative to the respective dilute monomer diffusivity), all data points for the Laf1-AK-Laf1 condensate converge onto a single line (Fig. S3). This observation suggests that the decrease in client diffusivity depends more strongly on the scaffold.

To assess any changes in the quaternary state of the peptide within the condensate, we dissolved the condensates after 1 hour of incubation by increasing the ionic strength and subsequently measured the diffusivity of free Aβ42-Atto565 in solution. This analysis yielded a diffusivity of 249 ± 12 µm^2^ s^−1^, which is consistent with the diffusivity of monomeric Aβ42 in solution measured for a dilute stock solution of Aβ42-Atto565. This result provides strong evidence that the peptide does not irreversibly aggregate within the condensate and can be recovered in monomeric form.

To prove that the peptide does not form reversible oligomers in the dense phase, we introduced Aβ42 peptides labelled with two different dyes (Atto565 and Atto647) into a phase separated solution of Laf1 and performed fluorescence cross-correlation spectroscopy (FCCS) in the condensed phase droplets. No cross-correlation signal was detected, indicating that the peptide remains monomeric in the dense phase (Fig. 4c). There remains a possibility of transient interactions between the peptides. Even if these interactions are short-lived, some cross-correlation signal would be expected as the peptides co-diffuse through the confocal volume. However, extremely rare transient interactions may remain undetected by FCCS.

Next, to investigate whether concentration-dependent diffusivity is a general trend for other client-scaffold systems, we repeated the experiments using Atto565 as the client for both the Laf1-AK-Laf1 and Ddx4(1-236) scaffolds. We observed similar trends of reduced diffusivity with increasing concentration for all analysed systems. Interestingly, for Ddx4(1-236), the diffusivity of the Atto565 client was measured to be as low as 0.02 µm^2^ s^−1^, which is 4 orders of magnitude lower than the bulk diffusivity of Atto565 (∼400 µm^2^ s^−1^). We verified the measured concentration-dependent diffusivities *via* FRAP experiments, which were consistent with FCS analysis (Fig. 4d and S4). For all the different client-scaffold systems, the measured diffusivity values as a function of client concentration could be fitted to a power law model of the form:

1 *D* = *KCγ*

where *D* is the measured diffusivity, *C* is the measured concentration, and *K* and *γ* are fitting parameters.^46^ The fitted parameters for our systems are reported in Table 1.

**Table 1:** Power law fitting parameters for recruitment of clients into scaffolds

Scaffold	Client	*K*	*γ*
Laf1-AK-Laf1	Aβ42-Atto565	236	−0.53 ± 0.02
Laf1-AK-Laf1	Atto565	861	−0.59 ± 0.09
Ddx4(1-236)	Atto565	656	−0.76 ± 0.06

Such a power-law relationship between diffusivity and concentration has previously been observed for self-diffusion in polymers^47,48^ and diffusion in porous crystalline materials.^49^ A possible explanation of the observed decrease in diffusivity of client molecules with increasing concentration (Fig. 4b) likely arises from the diffusion of client molecules in the porous structure of condensed phase scaffolds.^50^ At a concentration of 10 mM in the condensed phase, the average intermolecular distance is approximately 5 nm, resulting in ∼1 nm diameter pores. These dimensions are similar in size to the hydrodynamic radii of client molecules (between 0.5 and 1 nm in free solution). Increasing client concentration could block a fraction of the pores, leading to an observed decrease in diffusivity. Formally, the diffusivity in a porous material *D*_pore_ correlates with the bulk diffusivity, *D*_free_, according to the following relationship:^51^2
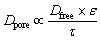
where *ε* is the porosity and *τ* is the tortuosity of the porous material. Furthermore, tortuosity follows a power law relationship with porosity:^52^

3 *τ* = *εk*

where *k* is a proportionality factor. As client concentration increases, we expect a reduction in both scaffold porosity and tortuosity within the scaffold, resulting in the observed decrease in effective diffusivity.

We note that the client concentration in the dense phase (micromolar) is significantly lower than the scaffold concentration (millimolar) and thus the client is unlikely to increase molecular crowding. The constant partitioning coefficient of the client as a function of client concentration indicates minimal cooperativity in client-scaffold interactions, implying their independence from client concentration. Therefore, the porous structure model offers the most plausible explanation for the observed decrease in diffusivity of the client with increasing client concentration, although further investigation into the underlying molecular mechanism is required.

## Conclusions

Phase separation of proteins generates distinct compartments with properties significantly different from the surrounding solution. Unsurprisingly, characterising such property changes is essential for understanding reaction and aggregation processes that occur within membrane-less organelles.^53^ Herein, we have introduced a new scanning FCS method, tl-FCS, which when combined with sineFCS can measure both concentration and diffusivity over several orders of magnitude. For a phase separated protein, this enables the analysis of both slowly diffusing molecules inside condensates and rapidly diffusing molecules in the surrounding dilute phase. Significantly, tl-FCS can be implemented without any hardware changes to standard confocal FCS systems. Leveraging the quantitative nature of sFCS, we successfully reconstructed the phase diagram of Ddx4(1-236), whilst being able to characterise properties within individual condensates. The ability to use small amounts of protein and preserve intra-condensate heterogeneity ensures significant advantage over existing methods. Indeed, methods based on time-integrated fluorescence are unable to quantify condensed phase concentrations since intensity does not scale predictably with concentration at high concentrations. Raman scattering can be used to probe the structure and composition of the condensed phase but is poorly suited for concentration measurements due to its sensitivity to molecular vibrations and the surrounding environment, which can vary significantly. While some Raman-based techniques have been used for concentration measurements, they almost always involve calibrations to account for such structural variations.^41^ Additionally, UV absorbance and nuclear magnetic resonance (NMR) measurements have been used to probe both dilute and dense phases.^54,55^ While dilute phase measurements are relatively straightforward, dense phase analysis is far more challenging since it represents less than 0.1% of the total protein volume. Many studies incorporate centrifugation in an attempt to coalesce condensed phase droplets so that sufficient sample can be collected for measurement.^56^ For example, NMR analysis in phase separation studies requires sample volumes of at least 0.5 ml, with protein concentrations typically between 0.5 and 1 mM.^44^ Obtaining such samples requires milligram quantities of purified protein, which can be difficult and costly. A further downside associated with coalescing individual condensates is that centrifugation homogenizes the condensed phase and thus any information regarding heterogeneity is irretrievably lost. Our sFCS measurements are performed using standard 384 well plates and require 3 orders of magnitude less protein solution per phase separation condition (20 µl *vs.* 0.5 ml). Several established FCS methods use laser scanning microscopes to measure molecular diffusion. Raster Image Correlation Spectroscopy (RICS) analyzes spatial 2D correlations in raster-scanned images to extract diffusion coefficients over wide fields of view.^57^ While powerful for mapping spatial heterogeneity, RICS requires careful calibration of the scanning parameters and is limited by the pixel dwell and line times inherent to raster scanning. Segmented FCS similarly segments conventional scanning data along lines to achieve better temporal resolution but also requires the use of external calibration solutions.^58^ In contrast, tl-FCS combines the advantage of self-calibrated scanning, with compatibility on commercial laser scanning microscopes. This positions tl-FCS as a versatile method that can capture fast diffusion dynamics without requiring external calibration. Further, sFCS allows measurements on a per-condensate level. Such granularity in measurements is simply not accessible to the existing traditional methods. It should be noted that due to the finite size of the excitation volume, FCS methods are able to measure the condensed phase inside droplets with diameters no smaller than 5 µm.

Investigation of the recruitment of client molecules in the scaffold revealed that client diffusivity is reduced by orders of magnitude when compared to data obtained by bulk measurements. Although a decrease in diffusivity with increasing concentration has been observed previously,^59^ the molecular origin of this behaviour remains unclear. Importantly, diffusivity decreases with increasing concentration in the dense phase following a power law relationship. This significant decrease in diffusivity could contribute to the observed stability of Aβ42 within biomolecular condensates despite the high local concentration.^15^ Indeed, monomeric peptide could further be recovered upon dissolving condensates after incubation, indicating that the decrease in diffusivity is not associated with the formation of irreversible aggregate species.

To conclude, we have presented a calibration-free method to study concentrations and diffusivities of molecules in phase separating systems, enabling the analysis of heterogeneity within individual condensates. Quantitative analysis of condensates will be helpful not only in basic research on molecular interactions and cellular organisation but also in understanding physiological functions^60^ and elucidating the role of condensates in a variety of diseases.^61–63^

## Materials and methods

### Setup for fluorescence correlation spectroscopy

Fluorescence correlation spectroscopy analysis was performed with a commercial point-scanning confocal microscope (Nikon C2i) with additional laser lines coupled for excitation. A continuous 561 ± 3 nm laser (Genesis MX 561-1000, Coherent, California, USA) was adjusted to a power output of 150 mW which was reduced to 4 mW with a ND filter. The laser was coupled to a C2si confocal laser scanning microscope (Nikon, Egg, Switzerland) set to a pinhole size of 20 µm. Phase separation samples were prepared in a MatriPlate 384 well plate with a cover slip bottom (Azenta Life Sciences, Berlin, Germany) with FCS measurements being performed with a 60X 1.2NA water immersion objective (Nikon, Egg, Switzerland). Photon emission was routed through an optical fibre, collimated with a fiber collimator (F950FC-A, Thorlabs, Bergkirchen, Germany), passed through a 625/52 emission filter (625/52 BrightLine, AHF, Tübingen, Germany) and focused with a doublet lens (AC254-050-A, Thorlabs, Germany) on a single photon counting module (SPCM-AQRH, Excelitas Technologies, Waltham, USA). The microscope was controlled with Nikon Elements C (Nikon, Egg, Switzerland) and the single photon data was collected with Symphotime64 (PicoQuant, Berlin, Germany), with both software running on separate computers connected in a home network. Photon data were analysed with custom MATLAB codes, using algorithms described in the SI.

### Protein expression and purification

#### Ddx4(1-236), Laf1-AK-Laf1 and Aβ42 expression

Ddx4(1-236), Abeta42 (Aβ42) and Laf1-AK-Laf1 were expressed and purified as previously described.^15,41,64^ Briefly, proteins were expressed recombinantly in *E. coli* BL21-GOLD (DE3) cells. Bacterial cultures were induced at OD 0.7 with 0.5 mM isopropyl d-thiogalactopyranoside (Bio Grade, PanReac AppliChem, Darmstadt, Germany) and grown for an additional 4 h (Aβ42) or 16 h (Ddx4(1-236), Laf1-AK-Laf1) at 37 °C.

For Aβ42, cells were spun down at 4500 rpm and re-suspended in working buffer (10 mM Tris, 1 mM EDTA buffer at pH 8.5). Inclusion bodies were recovered from lysed cells and solubilized upon addition of 8 M urea. Aβ42 was purified from the supernatant with a combination of ion exchange chromatography on a DEAE resin (GE Healthcare, Uppasala, Sweden) and size exclusion chromatography (SD 75 26/600, GE Healthcare, Uppasala, Sweden). The collected fractions were lyophilised and stored at −20 °C.

Ddx4(1-236) and Laf1-AK-Laf1 were purified by immobilized metal ion affinity chromatography (Chelating Sepharose, GE Healthcare, Uppasala, Sweden). Proteins were further purified by size exclusion chromatography using a size exclusion column (SD 75 16/600, GE Healthcare, Uppsala, Sweden) assembled on an ÄKTA Prime system (GE Healthcare, Uppsala, Sweden) using as eluent buffer 50 mM Tris at pH 7.5 and 500 mM NaCl (Laf1-AK-Laf1) or 1 M NaCl (Ddx4(1-236)). Protein stock was concentrated to 493 µM (Laf1-AK-Laf1) and 600 µM (Ddx4(1-236)) and aliquots (20 µl) were frozen and stored at −20 °C until use.

#### Aβ42 labelling

Aβ42 was tagged with Atto565 Dye (Atto-TEC GmbH, Siegen, Germany) by overnight incubation at 4 °C in the presence of a 3-fold molar excess of NHS ester dye, followed by purification with a size exclusion column (SD 75 16/600, GE Healthcare, Uppsala, Sweden) assembled on an ÄKTA Pure (GE Healthcare, Uppsala, Sweden) system. Final concentrations were assessed by measuring absorbance at 280 nm and 565 nm.

#### Ddx4(1-236) labelling

Ddx4(1-236) was expressed and purified with phosphate-buffered saline supplied with 1 M NaCl. Purified protein samples were tagged with Atto565 Dye (Atto-TEC GmbH, Siegen, Germany) by overnight incubation at room temperature in the presence of a 10-fold molar excess of NHS ester dye, followed by purification with a size exclusion column (SD 75 16/600, GE Healthcare, Uppsala, Sweden) assembled on an ÄKTA Pure (GE Healthcare, Uppsala, Sweden) system. Final concentrations were assessed by measuring absorbance at 280 nm and 565 nm.

### Phase separation experiments

All measurements were performed in a 384 well plate that was covered throughout the experiments. This setup effectively prevents significant evaporation during the measurement period. Additionally, we allowed the samples to equilibrate to room temperature before measurements and monitored for any instrumental thermal drift.

#### Phase diagram of Ddx4(1-236)

A stock solution of unlabelled Ddx4(1-236) was mixed with Ddx4(1-236) labelled with Atto565 in a concentration ratio of 750 : 1 to reduce the fluorescence intensity in the condensed phase. Phase separation was induced by diluting 2 µl of the mixed protein by 10 times in a sodium phosphate buffer (20 mM NaPi at pH 6.55 with varying concentrations of NaCl) in a 384 well plate. The phase separated droplets were allowed to settle by waiting for 15 minutes before performing FCS measurements.

#### Recruitment curves for Laf1-AK-Laf1

Phase separation was induced by diluting a 0.5 µl solution of Laf1-AK-Laf1 at 493 µM by 40 times in a sodium phosphate buffer (20 mM sodium phosphate pH 8 with 0.2 mM EDTA) in a 384 well plate. The phase separated droplets were allowed to settle by waiting for 15 minutes. Then a small volume of the client was added to the solution (0–1 µl, depending on the desired concentration) and allowed to equilibrate for 10 minutes. Finally, FCS measurements were performed.

#### Recruitment curves for Ddx4(1-236)

Phase separation was induced by diluting a 1 µl solution of Ddx4(1-236) at 615 µM in a Tris HCl buffer (10 mM Tris HCl at pH 7.5 with 100 mM NaCl) in a 384 well plate. The phase separated droplets were allowed to settle by waiting for 15 minutes. Then a small volume of the client was added to the solution (0–1 µl, depending on the desired concentration) and allowed to equilibrate for 10 minutes. Finally, FCS measurements were performed.

## Author contributions

P. M., S. S., P. A, conceived the idea, and S. S., P. A. and A. dM. supervised this project. P. M. carried out all the FCS experiments. M. P. carried out the expression, purification and labelling of all proteins used in this study. K. M., M. P. and P. M. analysed the data. P. M and P. A wrote the manuscript. S. S., P. A. and A. dM contributed to reviewing and editing the final manuscript.

## Conflicts of interest

There are no conflicts to declare.

## Supplementary Material

SC-017-D5SC05592J-s001

## Data Availability

The data supporting this article have been included as part of the supplementary information (SI). Supplementary information: details of data analysis methods and additional experimental data that further substantiate the conclusions of this work. See DOI: https://doi.org/10.1039/d5sc05592j.
