# Supplementary material for: Measuring concentration and diffusivity within biomolecular condensates using calibration-free scanning fluorescence correlation spectroscopy
Source: Chem Sci. 2025 Nov 19;17(2):985–95. doi: 10.1039/d5sc05592j (PMC12641978; doi:10.1039/d5sc05592j)
Supplement: SC-017-D5SC05592J-s001 [file SC-017-D5SC05592J-s001.pdf]

Supplementary Information (SI) for Chemical Science.
This journal is © The Royal Society of Chemistry 2025

Supplementary Information

Unravelling the heterogeneity of concentration and diffusivity within biomolecular condensates using scanning fluorescence correlation spectroscopy

Prerit Mathur[1], Marcell Papp[1], Katarzyna Makasewicz[1], Paolo Arosio[1], Andrew J. deMello[1]*, Stavros Stavrakis[1]*

[1]Institute for Chemical and Bioengineering, Department of Chemistry & Applied Biosciences, Eidgenössische Technische Hochschule (ETH Zürich), 8093, Zürich, Switzerland

Email: andrew.demello@chem.ethz.ch, stavros.stavrakis@chem.ethz.ch

Supplementary Text 1. Correction for photobleaching and sensor dead time

Due to the inherent behaviour of the sample and detection electronics, the collected data must be corrected for photobleaching of the sample (**Fig. S5**) and dead time of the electronics. The photobleaching was corrected for by assuming a bi-exponential decay of the raw photon intensity, $f_{decay}(t)$ [1]:

$$f_{decay}(t) = f_0 e^{-\frac{t}{t_1}} + f_1 e^{-\frac{t}{t_2}} \#(1)$$

where f_0, f_1, t_1 and t_2 are fitting parameters. The corrected photon signal thus can be extracted as:

$$F_{corrected}(t) = \frac{F(t)}{\sqrt{f_{decay}(t)/f_{decay}(0)}} + f_{decay}(0)\left(1 - \sqrt{f_{decay}(t)/f_{decay}(0)}\right) \#(2)$$

This corrected photon signal is then used for further analysis to calculate the autocorrelation function (ACF) (**Fig. S6**).

The dead time of the photodetector was accounted for with a method proposed by Neri et. al. [2,3]. For a measured photon count rate $\langle m \rangle$ and an expected count rate of $\langle n \rangle$, the detector dead-time t_d can be accounted for with the following equation:

$$\langle m \rangle = \frac{\langle n \rangle}{1 + \langle n \rangle \cdot t_d} \#(3)$$

The expected count rate was then used for further ACF calculations.

Supplementary Text 2. Data analysis methods

Point FCS algorithm:

For a photon signal $F(t)$, the autocorrelation function (ACF) with autocorrelation time τ is given by [4]:

$$G(\tau) = \frac{\langle F(t)F(t+\tau)\rangle}{\langle F(t)\rangle^2} - 1 = \frac{\langle \delta F(t)\delta F(t+\tau)\rangle}{\langle F(t)\rangle^2} \#(4)$$

Where $\langle F(t)\rangle$ is the average of $F(t)$ and the change in photon count $\delta F(t)$ is given by:

$$\delta F(t) = F(t) - \langle F(t)\rangle \#(5)$$

In the experiments described herein, the expected diffusion timescale of polymers and proteins is longer than the fluorophore triplet state lifetime (on the order of 1 µs). Thus, we performed autocorrelation analysis at timescales larger than 10 µs and used a theoretical model without triplet state dynamics. For 3D diffusion in a static gaussian confocal volume, the ACF, $G_D(\tau)$, can be modelled by:

$$G_D(\tau) = \rho \cdot \left(1 + \frac{\tau}{\tau_D}\right)^{-1} \cdot \left(1 + \frac{\tau}{S^2\tau_D}\right)^{-\frac{1}{2}} \#(6)$$

Here τ is the correlation time, τ_D is the diffusion time, S is the ratio of focal volume width to the focal volume height and ρ is the inverse of the number of molecules in the confocal volume.

Sinusoidal scanning FCS setup and algorithm

The sinusoidal scanning setup and algorithm are modified versions of previously published ultrafast scanning FCS [5]. In the current setup a focus tuneable liquid lens (FTL) (EL-16-40-TC-VIS-5D-C, Optotune, Dietikon, Switzerland) was introduced in the light path in a 4f-relay lens configuration[6]. The 4f configuration ensured that the magnification of the complete optical system (microscope and sinusoidal lens system) did not change when changing the focal length of the FTL. This modification was important to ensure that the scanning motion accurately represents the expected sinusoidal waveform. On applying a sinusoidal current

signal to the lens, the focal length also changes in a sinusoidal manner, allowing the confocal volume to scan through the sample. The amplitude of scanning depends on the applied current (**Fig. S7 and S8**).

For a scanning distance R, with the scanning lens running at a sinusoidal frequency f, the ACF can be modelled by:

$$G_s(\tau) = G_D(\tau) \times \exp\left[\frac{-R^2(\sin(\pi f \tau))^2}{4w_0^2\left(1+\frac{\tau}{S^2\tau_D}\right)}\right]\#(7)$$

Here $G_D(\tau)$ is the point FCS ACF as described previously, τ is the correlation time, S is the ratio of focal volume width to the focal volume height and ω_0 is the lateral size of the confocal volume.

Temporal Line scanning FCS setup and algorithm

Temporal line scanning FCS (tl-FCS) allows for calibration free fitting of ACF to measured experimental data. An advantage of tl-FCS over sinusoidal scanning method is that tl-FCS does not require any additional hardware. The laser scanner already available in a laser scanning confocal microscope can be used to scan across the sample needed to be measured. To model the ACF generated by tl-FCS, we start with a model for circular scan FCS[7]:

$$G_c(\tau) = G_D(\tau) \times \exp\left(\frac{-4R^2 \cdot \sin^2(\pi f \tau)}{w_0^2\left(1+\frac{\tau}{\tau_D}\right)}\right)\#(8)$$

where $G_D(\tau)$ is the point FCS ACF as described previously, τ is the correlation time, R is the radius of the scanning circle, f is the frequency of scan of the circle and τ_D is the characteristic diffusion time. To change from a circle to a line scan, we take the limit of a large radius ($R \to \infty$) where a circle becomes a straight line. Further, the scan speed (v) is assumed to be constant and $v = 2\pi R f$. Accordingly, the ACF becomes:

$$G_{tl}(\tau) = G_D(\tau) \times \exp\left(\frac{-v^2\tau^2}{w_0^2\left(1+\frac{\tau}{\tau_D}\right)}\right)\#(9)$$

Triplet state

In case triplet state dynamics need to be added to any ACF function described previously, the models can be simply adjusted as:

$$G_{triplet}(\tau) = G_i(\tau) \times \left[1 + T\left[\exp\left(\frac{-\tau}{\tau_{trip}}\right) - 1\right]\right]\#(10)$$

where T is the fraction of molecules in the triplet state, τ_{trip} is the triplet state lifetime and $G_i(\tau)$ is an autocorrelation function. For the triplet scenario, number of molecules in the confocal volume is modified to be $\frac{1}{\rho \cdot (1-T)}$.

Filtering of autocorrelation function

As the photon trace generated by line scanning is periodic but discontinuous due to defined start and end points of the scan path, the ACF calculated from the photon data has periodic ringing that makes interpretation of the data difficult (**Fig. S9 and S10**). Accordingly, the model $G_{tl}(\tau)$ cannot be used directly. Ringing was filtered using time shifted fluorescence correlation [8]. Briefly, the measured discontinuous photon trace ($I(t)$) was written as:

$$I(t) = M(t) \cdot V(t) \cdot F(t)\#(11)$$

Here $M(t)$ is the periodic function which represents the line scanning period, $V(t)$ is the change in fluorescence emission due to the modulation function (and is periodic with the same period as $M(t)$) and $F(t)$ is the fluorescence photon signal assuming continuous and static illumination of the sample. The autocorrelation of the measured photon trace with a lag time τ is given as:

$$\frac{\langle I(t)I(t+\tau)\rangle}{\langle I(t)\rangle^2} = \frac{\langle M(t)V(t)M(t+\tau)V(t+\tau)\rangle\langle F(t)F(t+\tau)\rangle}{\langle M(t)V(t)\rangle^2\langle F(t)\rangle^2}\#(12)$$

For a line scanning time-period T_p, we choose an N such that NT_p is much larger than any dynamic processes of interest happening in the sample, for instance $NT_p \gg \tau_D$, with τ_D being

the diffusion time of the sample of interest. Then, adding a lag time of NT_p to the autocorrelation in equation (12) results in:

$$\frac{\langle I(t)I(t+NT_p+\tau)\rangle}{\langle I(t)\rangle^2}=\frac{\langle M(t)V(t)M(t+NT_p+\tau)V(t+NT_p+\tau)\rangle\langle F(t)F(t+NT_p+\tau)\rangle}{\langle M(t)V(t)\rangle^2\langle F(t)\rangle^2}$$

(13)

It should be noted that here $M(t+NT_p+\tau)=M(t+\tau)$ and $V(t+NT_p+\tau)=V(t+\tau)$ because both $M(t)$ and $V(t)$ are periodic with a time-period T_p. Further, $F(t)$ is uncorrelated with $F(t+\tau)$ as NT_p is larger than any process in the sample, making $\langle F(t)F(t+NT_p+\tau)\rangle=\langle F\rangle^2$. This modification yields:

$$\frac{\langle I(t)I(t+NT_p+\tau)\rangle}{\langle I(t)\rangle^2}=\frac{\langle M(t)V(t)M(t+\tau)V(t+\tau)\rangle}{\langle M(t)V(t)\rangle^2} \#(14)$$

Now, dividing equation (12) by equation (14) results in:

$$\frac{\frac{\langle I(t)I(t+\tau)\rangle}{\langle I(t)\rangle^2}}{\frac{\langle I(t)I(t+NT_p+\tau)\rangle}{\langle I(t)\rangle^2}}=\frac{\langle F(t)F(t+\tau)\rangle}{\langle F(t)\rangle^2} \#(15)$$

Finally, the desired autocorrelation function is:

$$\frac{\frac{\langle I(t)I(t+\tau)\rangle}{\langle I(t)\rangle^2}}{\frac{\langle I(t)I(t+NT_p+\tau)\rangle}{\langle I(t)\rangle^2}}-1=\frac{\langle F(t)F(t+\tau)\rangle}{\langle F(t)\rangle^2}-1=G(\tau) \#(16)$$

This is identical to **Equation 4**. Thus, to filter the temporal line scan autocorrelation function, the autocorrelation from the measured photon trace must be divided by an autocorrelation function calculated from the same photon trace, but with an additional lag time. Next, knowing the scan speed, **Equation 9** can be used to calculate concentration and diffusivity.

Unlike line scanning used for membranes, which only works in scenarios when the diffusivity of the sample is slower than the scan speed [1], the current temporal line scanning can analyse rapidly diffusing species too as the complete time range of correlation times (down to 0.1

µs) are accessible. In our experiments, line scanning speeds of approximately 4–5 mm/s were employed when probing the dilute phase, where molecular diffusion is relatively fast. Conversely, for the lower molecular mobilities characteristic of the condensed phase, line scanning speeds were reduced to approximately 0.01 mm/s. These choices ensure that the scanning period matches the characteristic correlation times of the molecules under study, enabling precise measurements.

Supplementary Text 3. Comparison between UV, NMR and Raman based approaches

Protein phase separation can be characterized by several spectroscopic methods, each with distinct advantages and limitations. Table S1 compares various alternative methods to quantify protein phase separation, namely UV absorbance[9], Raman[10,11], and NMR[12].

Method	Sample requirement	Concentration range	Heterogeneity sensitivity
UV absorbance	Unlabelled, low to medium volume	µM to mM	Typically detects coalesced condensed phase; no information on molecular heterogeneity
Raman	Unlabelled, low volume	nM to µM	Provides conformational molecular information; lacks macroscale heterogeneity sensitivity
NMR	Unlabelled, medium to high volume	µM to mM	Provides information on molecular conformation; limited macroscale heterogeneity detection

Table S1 Comparison of UV absorbance, Raman, and NMR spectroscopy methods for quantifying protein phase separation.

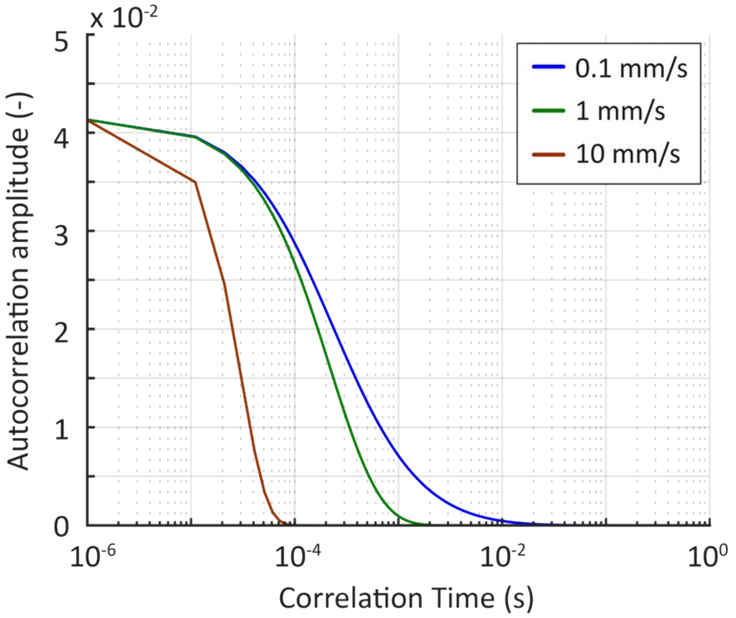

Fig. S1 Theoretical dependence of the ACF of tl-FCS on laser scanning speed for a sample with a diffusivity of $100\ \mu m^2/s$. The scan speed has its own characteristic time scale, which combined with the diffusion time scale of a molecule under investigation gives the overall correlation curve. Faster scanning leads to smaller characteristic decay times.

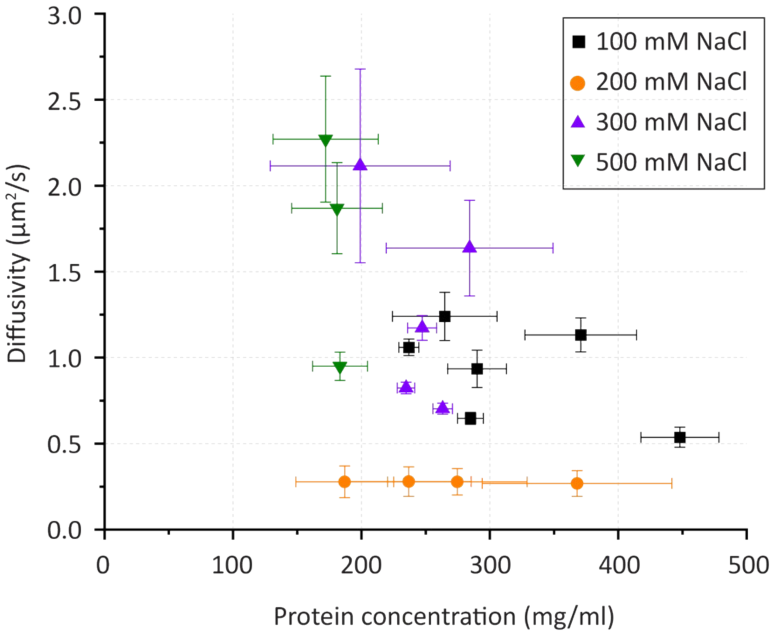

Fig. S2 Diffusivity distribution of individual condensates for condensed phase of Ddx4(1-236) at varying NaCl concentrations. As with concentrations (manuscript **Fig. 3**), the diffusivity values also show an intra-condensate heterogeneity reiterating the idea that protein condensates formed near to each other can have significant variations.

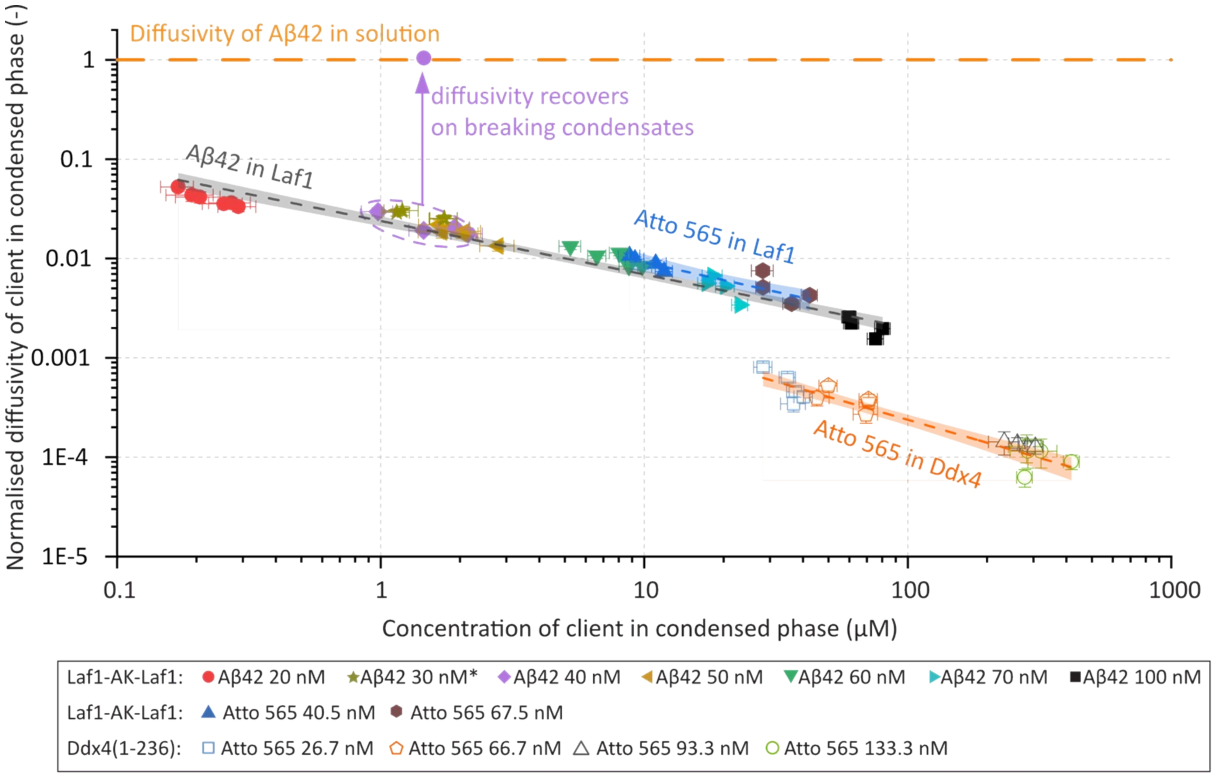

Fig. S3 Variation of the normalized diffusivity of the client molecules within the condensed phase as a function of client concentration for Aß42-Atto565 and Atto 565 recruited into Laf1-AK-Laf1 or Ddx4 droplets. Diffusivity values were normalized to 400 µm^2/s for Atto565 and 240 µm^2/s for Aß42-Atto565, corresponding to their respective diffusivities in the dilute phase. Solid straight lines indicate power-law fits of diffusivity versus concentration, with shaded areas denoting 95% confidence intervals. Upon condensate disruption by salt addition, the diffusivity of recovered Aß42-Atto565 matches that observed in the dilute phase. Error bars represent the standard error of replicate measurements (n=3.)

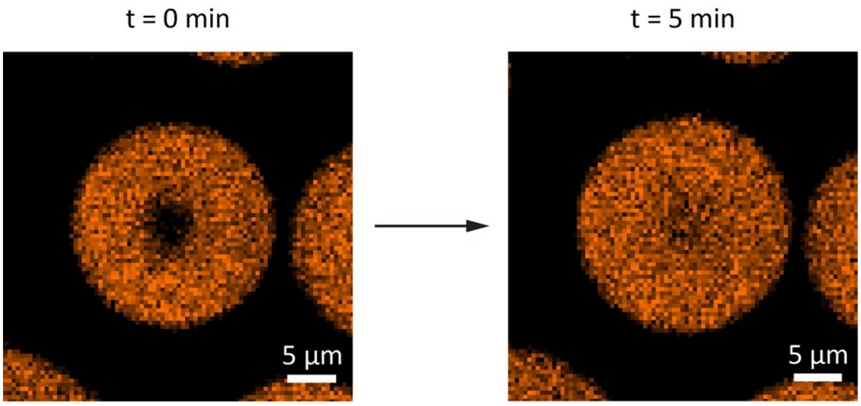

Fig. S4 FRAP analysis of Ddx4(1-236) condensates with Atto565 recruited into the condensates. A recovery time of more than 5 minutes for the bleached spot indicates the diffusivity to be on the order of $0.01\ \mu m^2/s$, which is consistent with FCS data.

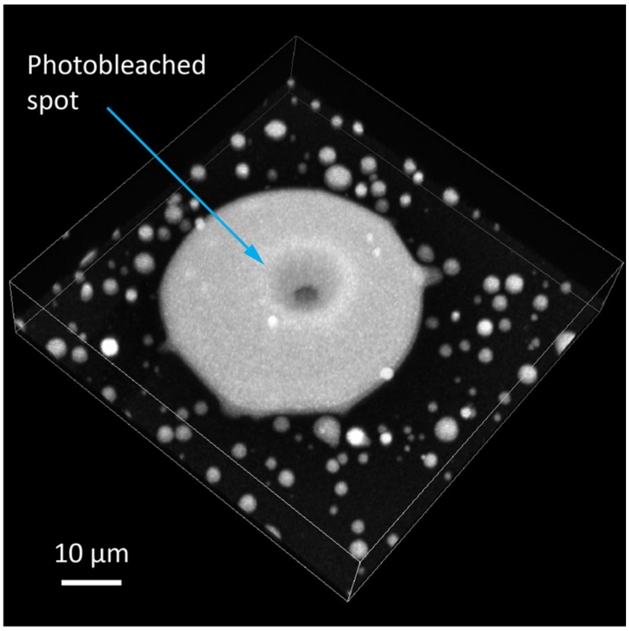

Fig. S5 Photobleached spot**.** Photobleached spot visible in confocal image of a condensed phase droplet of Ddx4(1-236) after point FCS measurement.

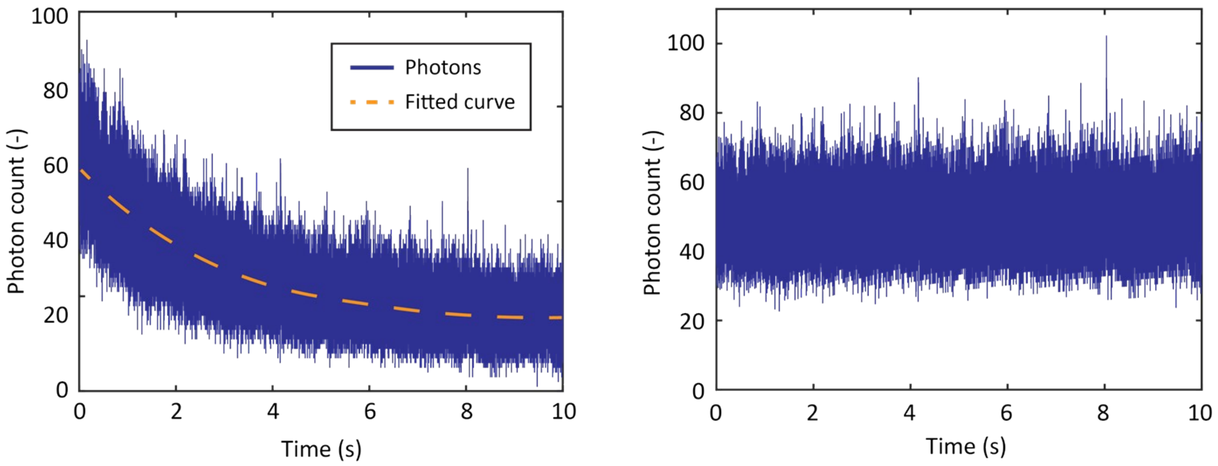

Fig. S6 Photobleaching correction. Collected photons show a decay in case the sample shows photobleaching. A bi-exponential decay is fit to the decay and inverting the fit function results in a photobleaching corrected photon trace.

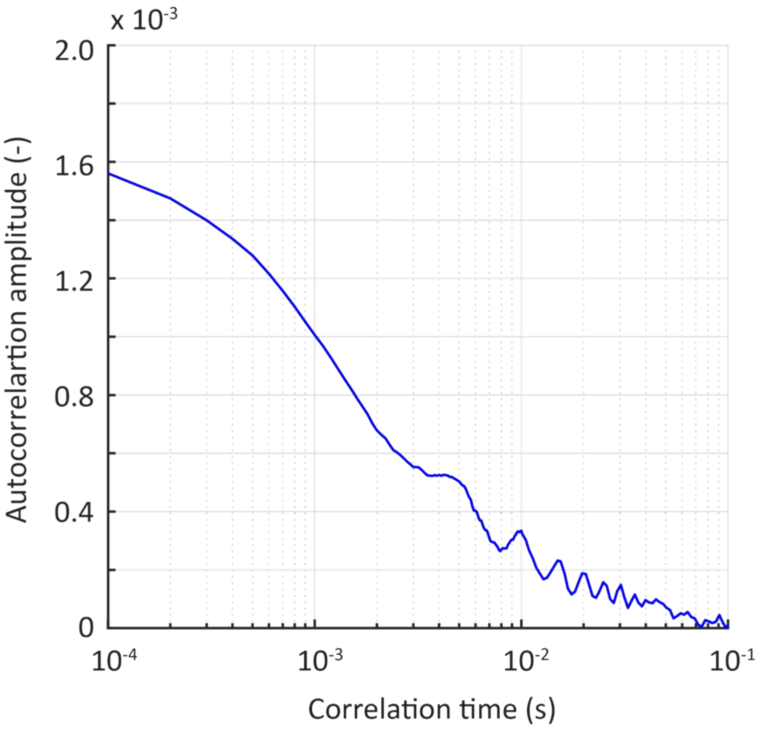

Fig. S7 Experimental sinusoidal scanning ACF for a condensed phase sample with a diffusivity of $4.2 \pm 0.4\, \mu m^2/s$. The scanning frequency was 200 Hz leading to periodic scan peaks from 5 ms onwards. The scanning peak amplitude is smaller as compared to **Fig. S8** as the ACF has already decayed due to high diffusivity.

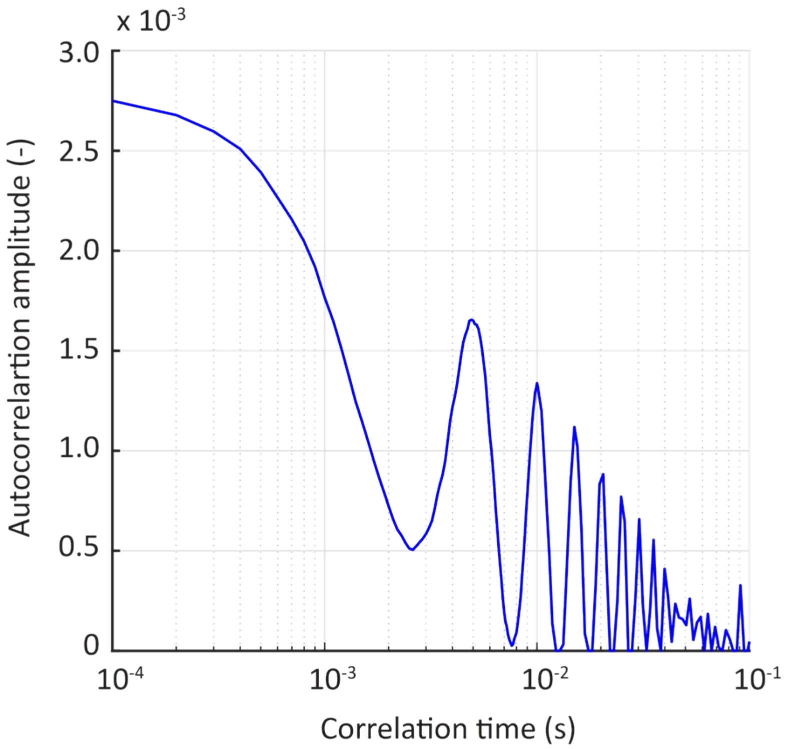

Fig. S8 Experimental sinusoidal scanning ACF for a condensed phase sample with a diffusivity of $0.35 \pm 0.03\ \mu m^2/s$. The scanning frequency was 200 Hz leading to periodic scan peaks from 5 ms onwards. The scanning peak amplitude is higher than Fig. S7 since the ACF takes longer to decay due to a low sample diffusivity.

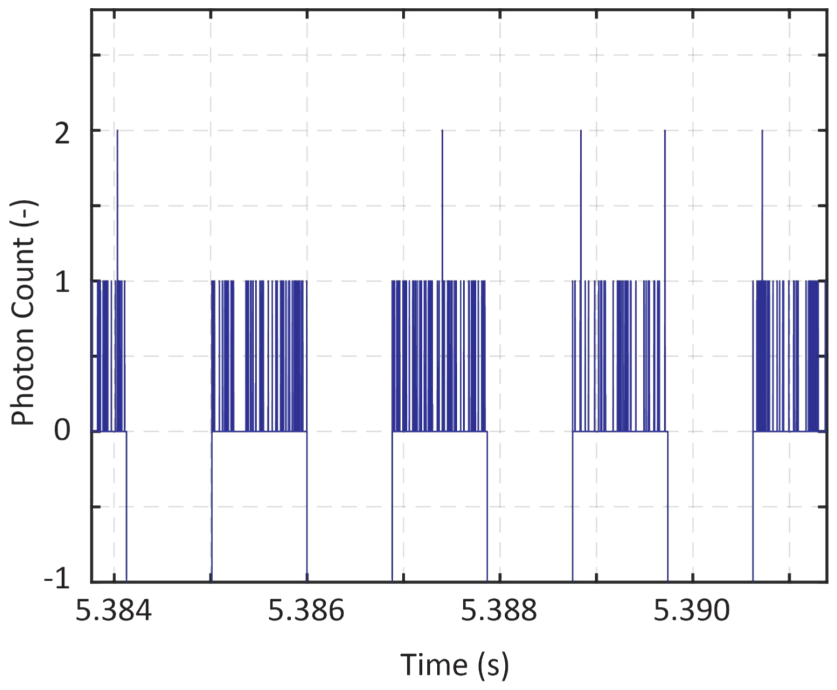

Fig. S9 Discontinuous nature of line scanning. The forward line scan has positive (>= 0) detected photons. Any photons collected during the traceback portion are assigned negative values to be ignored by the autocorrelation algorithm.

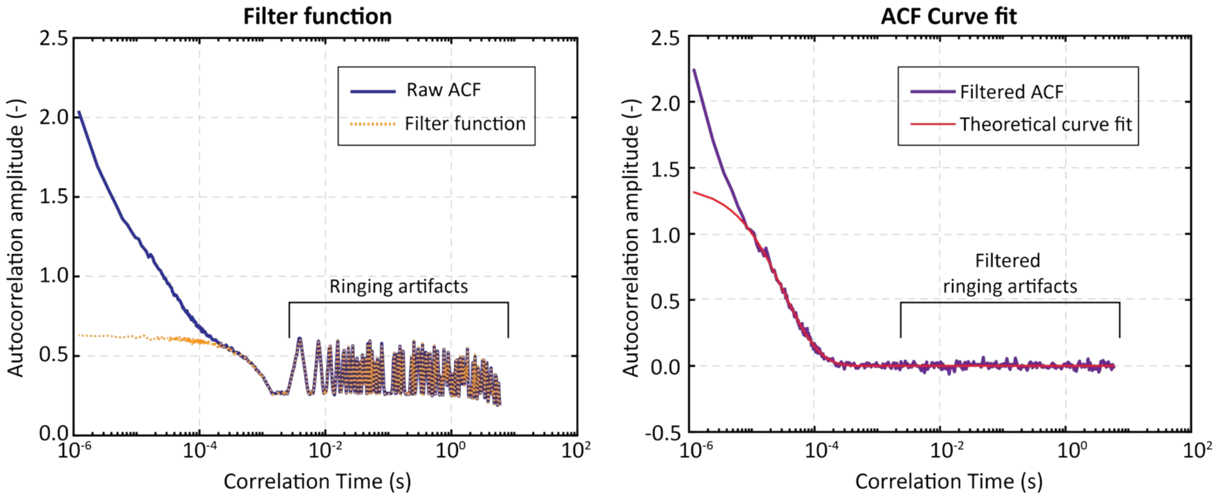

Fig. S10 Filtering of ringing artifacts**.** The ACF of a discontinuous line scan has ringing artifacts which can be suitably filtered out to create a smooth ACF function, which can be fit with theoretical models. Here the filtering process is shown for Atto 565 dye. The fitting function used is for 3D diffusion without triplet state contribution.

References

1 J. Ries, S. Chiantia and P. Schwille, *Biophys. J.*, 2009, **96**, 1999–2008.

2 L. Neri, S. Tudisco, F. Musumeci, A. Scordino, G. Fallica, M. Mazzillo and M. Zimbone, *Rev. Sci. Instrum.*, 2010, **81**, 086102.

3 E. Schaub, *Opt. Express*, 2013, **21**, 23543–23555.

4 J. R. Lakowicz, *Principles of Fluorescence Spectroscopy*, Springer, 3rd edn., 2006.

5 M.-T. Wei, S. Elbaum-Garfinkle, A. S. Holehouse, C. C.-H. Chen, M. Feric, C. B. Arnold, R. D. Priestley, R. V. Pappu and C. P. Brangwynne, *Nat. Chem.*, 2017, **9**, 1118–1125.

6 F. O. Fahrbach, F. F. Voigt, B. Schmid, F. Helmchen and J. Huisken, *Opt. Express*, 2013, **21**, 21010–21026.

7 Z. Petrášek, J. Ries and P. Schwille, in Methods Enzymol., ed. N. G. Walter, Academic Press, 2010, vol. 472, pp. 317–343.

8 G. Persson, P. Thyberg and J. Widengren, *Biophys. J.*, 2008, **94**, 977–985.

9 J. P. Brady, P. J. Farber, A. Sekhar, Y.-H. Lin, R. Huang, A. Bah, T. J. Nott, H. S. Chan, A. J. Baldwin, J. D. Forman-Kay and L. E. Kay, *Proc. Natl. Acad. Sci. U.S.A.*, 2017, **114**, E8194–E8203.

10 I. López-Peña, B. S. Leigh, D. E. Schlamadinger and J. E. Kim, *Biochemistry*, 2015, **54**, 4770–4783.

11 R. Sun, Y. Zhuang, Y. Lin and F. Hu, *Nat. Commun.*, 2025, **16**, 8552.

12 G. Wider and L. Dreier, *J. Am. Chem. Soc.*, 2006, **128**, 2571–2576.
